# Resveratrol Enhances Neuroplastic Changes, Including Hippocampal Neurogenesis, and Memory in Balb/C Mice at Six Months of Age

**DOI:** 10.1371/journal.pone.0145687

**Published:** 2015-12-22

**Authors:** Mario Torres-Pérez, Ruth Ivonne Tellez-Ballesteros, Leonardo Ortiz-López, Muhammad Ichwan, Nelly Maritza Vega-Rivera, Mario Castro-García, Ariadna Gómez-Sánchez, Gerd Kempermann, Gerardo Bernabe Ramirez-Rodriguez

**Affiliations:** 1 Laboratory of Neurogenesis, Division of Clinical Investigations, National Institute of Psychiatry “Ramón de la Fuente Muñiz”, Calz. México-Xochimilco 101, 14370, México, D.F., México; 2 CRTD - Center for Regenerative Therapies Dresden, Tatzberg 47–79, 01307, Dresden, Germany; 3 Department of Pharmacology and Therapeutic, Faculty of Medicine, Universitas Sumatera Utara, Jalan Dr. Mansur 5, Medan, Indonesia; 4 Laboratory of Neuropsychopharmacology, Division of Neurosciences, National Institute of Psychiatry “Ramón de la Fuente Muñiz”, Calz. México-Xochimilco 101, 14370, México, D.F., México; 5 DZNE, German Center for Neurodegenerative Diseases, Dresden, Tatzberg 47–49, 01307, Dresden, Germany; Case Western Reserve University School of Dental Medicine, UNITED STATES

## Abstract

Resveratrol (RVTL) is a flavonoid found in red wine and has been publicized heavily as an anti-aging compound. Indeed, basic research confirms that although there is much hype in the promotion of RVTL, flavonoids such as RVTL have a wide range of biological effects. We here investigated the effects of RVTL treatment on hippocampal plasticity and memory performance in female Balb/C mice, a strain with low baseline levels of adult neurogenesis. Two weeks of treatment with RVTL (40 mg/kg) induced the production of new neurons in vivo by increasing cell survival and possibly precursor cell proliferation. In addition, RVTL decreased the number of apoptotic cells. The number of doublecortin (DCX)-expressing intermediate cells was increased. RVTL stimulated neuronal differentiation in vitro without effects on proliferation. In the dentate gyrus, RVTL promoted the formation and maturation of spines on granule cell dendrites. RVTL also improved performance in the step down passive avoidance test. The RVTL-treated mice showed increase in the levels of two key signaling proteins, phospho-Akt and phospho-PKC, suggesting the involvement of these signaling pathways. Our results support the vision that flavonoids such as resveratrol deserve further examination as plasticity-inducing compounds in the context of successful cognitive aging.

## Introduction

In the context of healthy successful aging, there is hope that natural compounds would promote or at least help to stabilize neural plasticity and thus contribute to maintaining cognitive function in old age. It has been proposed and popularized that certain natural compounds have direct positive effects on neuronal plasticity [1, 2, 3].

Flavonoids have received prominent attention in this context [4–6] and among these RVTL has been much publicized, presumably because it is found in red grape skin and hence red wine, thus (at least at face value) becoming a plausible single-factor mediator of the positive effects of the mediterranean diet on aging [7, 8]. However, several clinical studies with RVTL could not confirm relevant beneficial effects. Nevertheless, RVTL remains neurobiologically interesting, for example because of its anti-aging effects through the deacetylase sirtuin-1 (Sirt1) [9].

The clinical evidence is also not terminally conclusive, because many important questions are open. Among these are the relevant biological targets of the intervention and the related question of how a suitable group of subjects would have to be stratified to observe an effect of RVTL [10]. After all, the acute treatment within the study context is different from the potentially essentially lifelong intake in the context of the mediterranean diet.

Resveratrol is a phytoalexin found in grapes, raspberries, mulberries and peanuts [9] and exists in both *cis*- or *trans*-forms. *Trans*-RVTL has anti-cancer and anti-inflammatory effects [11–13]. RVTL crosses the blood brain barrier [14], where RVTL reverted deficits in spatial memory after stroke [15] and acted as a neuroprotective agent [16]. Finally, treatment with RVTL extended the life span [9] and in addition to its interaction with Sirt-1, which suggests a mechanism involving epigenetic changes, the effects of RVTL also involved signaling pathways such as the AMP-activated protein kinase (AMPK) [17, 18].

Our general hypothesis is that adult hippocampal neurogenesis provides “neurogenic reserve” that allows for the maintenance of cognitive flexibility in older age [19]. Given the presumed profile of bioactivity of RVTL an effect on neural precursor cells and adult hippocampal neurogenesis seems likely. However, previous studies indicated that RVTL would decrease adult hippocampal neurogenesis and spatial memory in young C57Bl/6 mice [20], and reduce proliferation of neural precursor cells from the adult hippocampus [21]. In contrast, positive effects of RVTL on neurogenesis were seen in prenatally stressed rats [22] and in a mouse model of chronic fatigue [23] as well as in a knockout for the calcitonin gene related peptide (CGRP) [24]. Long-term treatment with RVTL prevented age-related cognitive decline and was associated with maintained greater level of adult neurogenesis [25]. In that study, the surprising aspect was that only four weeks of treatment in late mid-life was reported to have very long-term effects into old age.

In the present study we investigated six month old rather than younger mice, using a strain of mice (Balb/C) as a genetically defined cohort different from previous studies and with low baseline levels of adult neurogenesis [26], and applied RVTL for 14 days. We chose a protocol, in which RVTL was applied after labeling of hippocampal precursor cells with BrdU, so that the effect of RVTL on the survival of newborn cells rather than the proliferation of the precursor cells was in the center of interest. At the age of 6 months physiological levels of adult neurogenesis have declined to very low baseline levels [27, 28].

We thus hypothesized that RVTL might be effective in influencing structural hippocampal plasticity by regulating adult hippocampal neurogenesis and dendritic spines in a 14 days treatment model in female Balb/C mice at six months of age and improved behavioral performance. Balb/C mice were chosen because they show medium to low levels of baseline adult hippocampal neurogenesis but high relative numbers of surviving newborn cells in comparison to CD1 mice [26] and at least in one study was observed to have a very large sensitivity to activity-induced regulation [29]. Our previous work on the effects of melatonin on adult hippocampal neurogenesis had found strong effects on the survival of newborn neurons in this strain of mice [30].

## Materials and Methods

### Animals and ethics statement

One hundred two month-old female Balb/C mice were obtained from Harlan (México, D.F. México). Animals were housed in standard laboratory cages (12-hour light/12-hour dark cycles at a temperature of 23 ± 1°C) following the institutional and legal regulations regarding to animal ethics and handling. Mice had access to food and water *ad libitum* and were left to acclimatize in their environment until they reached the age of 6 months. The light/dark cycle corresponded to the timing of lights on (Zeitgeber time 0; ZT0) at 0700 hours (h) and to the timing of lights off (Zeitgeber time 12; ZT12) at 1900 h. All institutional and legal regulations regarding animal ethics and handling were followed for the *in vivo* experiments (Ethical Committee for Research of the National Institute of Psychiatry approved the present study with the number IACUC SIC092025). In our study we decided to use female instead of male mice based on reports of increased aggression in rodent males, while this was not observed in females [31].

### Experimental design, RVTL treatment and BrdU injections

**Fig 1:**
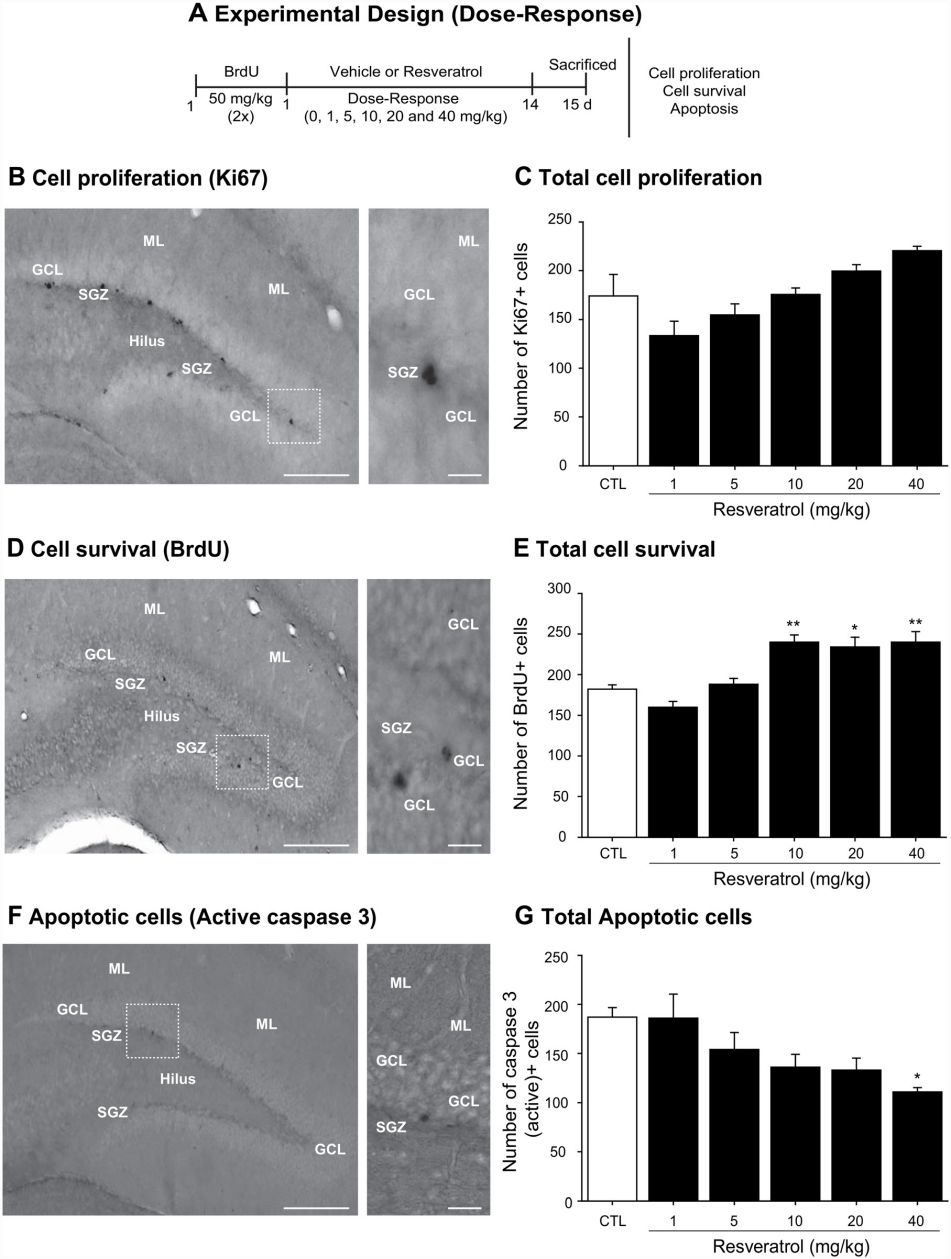
Resveratrol promotes cell survival in the hippocampus of female Balb/C mice at six months of age. (**A)** Experimental design. Balb/C female mice were kept under normal housing conditions until the age of 6 months. Mice were treated with vehicle or resveratrol (RVTL; 1, 5, 10, 20 and 40 mg/kg) during 14 days to evaluate its effect on key events of the hippocampal neurogenic process: cell proliferation, cell survival and on the doublecortin-associated new neurons. (**B)** Representative micrograph of proliferative cells expressing Ki67 is shown. (**C)** Ki67-labeled cells were not significantly changed in mice treated with RVTL. Panel (**D)** shows a representative micrograph of survival newborn cells identified by BrdU-labeling. (**E)** Quantification of BrdU-labeled cells show significant increase in cell survival in mice treated with 10, 20 and 40 mg/kg of RVTL (**p = 0.005, *p = 0.017, respectively). **F)** Representative micrograph of cells expressing caspase 3 (active) is shown. (**G)** Apoptotic cells show significant decrease in mice treated with 40 mg/kg of RVTL (*p = 0.043). However, lower doses of RVTL (1, 5, 10 and 20 mg/kg) did not significantly decrease the absolute number of caspase 3 (active)-labeled cells (*p* = 1, *p* = 0.94, *p* = 0.17, *p* = 0.14, respectively). Data are expressed as the mean ± S.E.M. of the absolute number of cells. Bonferroni’s post hoc test was performed after one-way ANOVA for cell proliferation, survival and apoptotic cells. Micrographs also show the granule cell layer (GCL), molecular layer (ML), subgranular zone (SGZ) and the hilus (H). Scale bar = 160 μ m. Scale bar in amplification of panels B, D and F = 30 μm. *n* = 4 mice per group.

Once mice had reached 6 months of age, they received two injections of BrdU (50 mg/kg; Sigma, St. Louis, MO, USA) 2 h apart (Fig 1A). Then, the animals were treated with RVTL (Sigma, St. Louis, MO, USA) in concentrations (0.0044, 0.022, 0.044, 0.088, 0.176 mg/ml) to yield different doses (1, 5, 10, 20 or 40 mg/kg of body weight) per day for 14 days. The estimated daily RVTL intake for each mouse was based on an average daily water consumption rate of 5 ml per day. Resveratrol was dissolved in minimum absolute ethanol and then added to drinking water at a final ethanol concentration of 0.3%. Fresh RVTL solution was prepared every third day and provided in feeding bottles that were protected from light. Mice in the control groups received water containing 0.3% of ethanol. The water consumption was similar for all experimental groups. Control- and RVTL-treated mice were housed under normal conditions in groups of 4 animals.

### Brain dissection and tissue processing for immunohistochemistry

One day after the end of the treatment, mice were sacrificed by an overdose of ketamine and perfused transcardially with 4% *p*-formaldehyde in 0.1 M phosphate buffer (pH 7.4). Brains were removed and post-fixed for 24 h before they were stabilized in 30% sucrose in phosphate buffer [32]. Brains were cut into 40 μm coronal sections on a tabletop sliding microtome (Leica, Buffalo Grove, IL, USA). The sections were stored at 4°C in cryoprotectant solution and stained following the free floating immunohistochemistry method and pretreated for BrdU-immunodetection by incubation in 2 N HCl for 30 minutes at 37°C followed by 2 washes in 0.1 M borate buffer (pH 8.5) for 10 minutes each [30, 32].

### Immunohistochemistry and cell quantification

To analyze cell proliferation, survival and the immature neurons in the subgranular or in the granular cell layer of the dentate gyrus, we identified specific markers of each step with the peroxidase method, as described previously [32]. In addition, we analyzed the apoptotic cells in the granular cell layer.

Cell proliferation was determined with a rabbit anti-Ki67 antibody (1:2000; Abcam, San Francisco, CA, USA), survival of newborn cells with a rat anti-BrdU antibody (1:500; Accurate, Westbury, NY, USA), the immature neurons with a goat anti-doublecortin (DCX) antibody (1:200; Santa-Cruz Biotech, Santa Cruz, CA, USA) and apoptotic cells with a rabbit anti-caspase-3 (active) antibody (1:100; Merck-Millipore, México, D.F. México). Secondary biotinylated anti-rabbit, anti-rat and anti-goat antibodies were from Jackson Immunoresearch (West Grove, PA, USA). Sections were clarified and mounted in Neomount medium (Merck, Whitehouse Station, NJ, USA).

The number of Ki67-, BrdU-, DCX- or caspase-3 (active)-labeled cells was determined in series of every 6^th^ section from all animals. Positive cells were counted using a 40x objective throughout the rostro-caudal extent of the granule cell layer on a light microscope coupled to a camera (Leica, Buffalo Grove, IL, USA). Counting was done as described previously, with a modified version of the optical fractionator. The cells appearing in the uppermost focal plane were not counted in order to avoid oversampling [32]. The estimated total number of labeled cells per granular cell layer was calculated from estimated resulting numbers multiplied by 6.

### Phenotypic analysis

Newly mature formed neurons in the dentate gyrus of adult mice were analyzed in one-in-twelve series of sections from animals of control- or RVTL (40 mg/kg)-groups by immunofluorescent double staining [32]. From the percentages of every phenotype and to the total number of BrdU-labeled cells the total number per phenotype was calculated. The newborn neurons were identified by the BrdU/NeuN co-labeling. Analysis was performed by confocal microscopy (Zeiss LSM 510 Meta; Thornwood, NY, USA) in sequential scanning mode to avoid cross-bleeding between channels. Double-labeling was confirmed by three-dimensional reconstructions of z-series covering the entire nucleus (or cell) in question. The primary antibodies were: monoclonal mouse anti-NeuN (1:100; Chemicon, Temecula, CA, USA) and rat anti-BrdU antibody (1:250; Accurate, Westbury, NY, USA). Fluorophore-coupled secondary antibodies were: anti-rat TRITC or anti-mouse FITC. All secondary antibodies were raised in donkey and diluted 1:250 (Jackson Immunoresearch, West Grove, PA, USA). Sections were mounted in polyvinyl alcohol with diazabicyclo-octane as anti-fading agent (PVA-DABCO).

### Hippocampal neurosphere cultures

Precursor cells were isolated from the adult dentate gyrus and collected into neurosphere medium consisting of mouse DMEM/F12 basal medium (Gibco, Life Technologies, Karlsruhe, Germany) plus mouse NeuroCult NSC Proliferation Supplements (Stem Cell Technologies, Grenoble, France) with 2% bovine serum albumin (Gibco, Life Technologies, Karlsruhe, Germany), 2 μg/ml heparin (Sigma-Aldrich, Munich, Germany). Culture medium was complemented with 20 ng/ml purified mouse receptor-grade epidermal growth factor (EGF; BD Biosciences, Heidelberg, Germany) and 10 ng/ml recombinant bovine FGF-2 (Roche, Mannheim, Germany). Cells were incubated in humidified 5% CO_2_ for 12 d to permit neurosphere formation in the presence of RVTL (0, 0.001, 0.01, 0.1, 1 or 10 μM).

### Adherent adult hippocampal precursor cell cultures

For adherent adult hippocampal neural precursor cell cultures, cells were isolated as previously reported [33, 34]. After the brains were removed from the skull, the dentate gyrus was dissected and dissociated by enzymatic digestion. The resulting cell suspension was separated by centrifugation using a Percoll gradient. Precursor cells were plated on laminin pre-coated coverslips or 96 multi-well plates and cultured with 20 ng/ml of human Epidermal Growth Factor (EGF) and 20 ng/ml of human Fibroblast Growth Factor-2 (FGF2; both from PeproTech, México, D.F. México) in Neurobasal medium supplemented with B27 (Gibco, Life Technologies, Grand Island, NY, USA) for 1 day followed by 2 additional days in the presence of growth factors plus RVTL (0, 0.001, 0.01, 0.1, 1 or 10 μM). Cell proliferation was determined by manual quantification of BrdU immunocytochemistry in an epifluorescence microscope Nikon Eclipse Ti U (Nikon, Melville, NY, USA).

For differentiation studies, adherent precursor cells were cultured in medium containing EGF and FGF2 for 1 day followed by 2 additional days in the presence of growth factors plus RVTL (0, 0.001, 0.01, 0.1, 1 or 10 μM) before cells were switched to a medium containing only RVTL (0, 0.001, 0.01, 0.1, 1 or 10 μM) for 5 days. Culture media was replaced every third day. Cell survival and the phenotype of differentiated precursor cells were assessed by quantitative immunocytochemistry. Positive cells were identified with specific primary antibodies: rabbit anti-GFAP (1:2000; Dako, Via Real, CA, USA); mouse anti-βIII-tubulin (1:2000; Promega, Madison, WI, USA) or monoclonal anti-MAP2 (1:500; Sigma, St. Louis, MO, USA).

### Golgi-Cox impregnation

For metal impregnation, brains were removed from the skull and immersed in a Golgi-Cox solution during 21 days. Every third day, the Golgi-Cox solution was replaced with fresh solution. Brains were transferred to a 30% sucrose solution and sliced on a table-top sliding microtome (Leica). Coronal brain sections (200 μm) were collected in 3% sucrose solution and mounted onto pre-gelatinized glass slides. Golgi-Cox impregnation was revealed with 18.7% ammonia solution and fixed in Kodak rapid fix (Kodak, Rochester, NY, USA). Brain slices were dehydrated and clarified in Neoclear before they were coverslipped with Neomount medium (Merck, Whitehouse Station, NJ, USA) [35, 36]. Twenty Golgi-stained neurons in the granule cell layer (GCL) of the dentate gyrus were analyzed to determine dendritic spine density in every 10 μm of secondary dendrites of rodents exposed to either control- or RVTL (40 mg/kg). Control- and RVTL-treated mice were housed under normal conditions in groups of 4–5 animals.

### Behavioral experiments

#### Step-down passive avoidance test (SDPA)

Sixteen mice were used in the SDPA test (8 mice per group). This test has two phases. During the training phase the animal is punished for making the instinctive response of moving from a brightly lid chamber into a darkened one [37]. After a specified delay interval, the rodent is tested under the same conditions to determine the length of time, after which the animal moves into the dark side of the chamber (test phase). The latency to repeat the punished response is considered as an index of memory. Here, we used an automated testing apparatus (Ugo Basile; Comerio, VA, Italy) located in a well-illuminated room. One day after the end of the treatment, the training in the SDPA was performed (day 15). Mice were subjected to a foot-shock (0.4 mA, 5 s) as soon as they completely descended onto the grid floor of the dark side of the apparatus. Retention tests without shocks were performed 1.5 (day 15) and 24 h (day 16). A researcher blind to the experimental groups analyzed the parameters.

#### Radial arm maze test (8-RAM)

Twelve mice per group (control versus 40 mg/kg of RVTL) were used to analyze their behavioral performance in the 8-RAM test. One day after the end of the treatment, we started the training of mice to find food cereal chocolate pellets in four randomly selected arms of the maze that was elevated 45 centimeters above the floor. Training was performed during 15 days. All experiments were performed in a well-illuminated room containing several visual cues to aid in spatial localization. Mice were trained once a day and sessions were continued until the mouse had collected all cereal-chocolate pellets, at the latest after 10 minutes. After each trial, the 8-RAM was carefully cleaned with antiseptic solution containing ethanol (70%). All trials were video recorded with a Canon Power Shot SX50 HS (Canon, Japan) and analyzed by a researcher blind to the experimental condition. The parameters analyzed were: 1) working memory errors (entries into baited arms that had already been visited during the same trial), 2) reference memory errors (entries into unbaited arms), and 3) the locomotor activity (total number of entries).

### Western blot for key signaling protein levels in the hippocampus

Hippocampi from control- or RVTL-treated mice were dissected and lysed as reported previously [35, 36]. Control- and RVTL-treated mice were housed under normal conditions in groups of 4–5 animals. The lysate was obtained in RIPA buffer (150 mM NaCl, 10% glycerol, 0.5 mM EDTA, 0.5% triton X-100, 1 mM PMSF, 25 μg/ml leupeptin, 25 μg/ml aprotinin and 1 mM sodium ortho-vanadate in 50 mM Tris-HCl, pH 7.6), homogenized with an ultrasonic homogenizer for 30 seconds and centrifuged at 3300 xg for 15 minutes. Total protein content was quantified using Bradford reagent (Bio-Rad, Philadelphia, PA, USA). Protein separation was performed by the Laemmli method [38] and transferred to PVDF or nitrocellulose paper. Membranes were blocked with 5% skim milk in 0.05% Tween 20-TBS (TTBS) and incubated with rabbit-anti-phospho Akt or rabbit anti-Akt (Cell Signaling, Danvers, MA, USA); mouse monoclonal anti-phospho-p44/42 MAP kinase or rabbit anti-p44/42 MAP kinase (Cell Signaling); rabbit anti-phospho-CREB or rabbit anti-CREB (Cell-Signaling); rabbit anti-phospho-PKC (Cell Signaling). The mouse anti-GAPDH antibody (Chemicon, Temecula, CA, USA) was used for the loading control. Blots were washed 3 times with TTBS and incubated for 1 hour in a 1:3000 dilution of the appropriate secondary antibody: phosphatase-conjugated donkey anti-mouse; donkey anti-goat, or donkey anti-rabbit. Proteins were visualized with the enhanced chemiluminescence detection system (Merck-Millipore). Images were scanned and densitometry analysis was performed with the ImageJ software (NIH, Bethesda, MA, USA).

### Statistical Analysis

Analysis was performed using SigmaStat 3.1 software. The results are presented as mean ± standard error of the mean SEM. For *in vivo* cell proliferation, survival and apoptosis mean differences between groups were analyzed using one-way ANOVA followed by Bonferroni’s post-hoc test, where appropriate. Doublecortin-labeled cells were analyzed using a Kruskal-Wallis-ANOVA on ranks followed by Student-Newman-Keuls comparison because the equal variance test failed. Mature new neurons, dendritic spines number, proportions of dendritic spines and relative protein levels were analyzed by unpaired t-test. The results of the hippocampal dependent behavior (SDPA-test) and the results of 8-RAM test were analyzed with a repeated measures two-way ANOVA, both followed by the appropriate post-hoc test. Two-tailed unpaired t-tests were used for comparison between two groups. The results derived from precursor cells were analyzed using one-way ANOVA followed by Tukey post-hoc test. Differences were considered statistically significant at *p* ≤ 0.05.

## Results

### Resveratrol affects cell survival and doublecortin-cells without affecting cell proliferation in vivo

First, we determined whether RVTL might affect cell survival of BrdU pre-labeled cells (Fig 1D and 1E). After 14 days of treatment RVTL favored the survival of newborn cells in the dentate gyrus of mice at six months of age as indicated by an increase in the number of BrdU-labeled cells compared with the control group (Fig 1E). The number of surviving cells in the dentate gyrus of mice treated with the highest doses of RVTL (10, 20 or 40 mg/kg) reflected an increase of 31%, 28%, 31%, respectively (control: 182 ± 5.3; RVTL 10 mg/kg: 240 ± 8.83; RVTL 20 mg/kg: 234 ± 12; RVTL 40 mg/kg: 240 ± 13; F_5,23_ = 12.28; *p* = 0.005, 0.017, 0.005, respectively). However, the lowest dose of RVTL caused a decrease in cell survival without reaching statistical significance (RVTL 1 mg/kg: 160 ± 7.2; F_5,23_ = 12.28; *p* = 0.79). To rule out that the pro-survival effect of RVTL might be only a consequence of an increased cell proliferation, we also analyzed Ki67-expression as an intrinsic proliferation marker (Fig 1B and 1C). Quantification at 14 days after treatment revealed that the dose of 1 mg/kg decreased the absolute number of proliferative cells in the dentate gyrus without reaching statistical significance (control: 174 ± 22.04 versus RVTL 1 mg/kg: 133 ± 14.77; F_5,23_ = 6.12; *p* = 0.17; Fig 1B and 1C). In contrast, the highest doses of RVTL (20 and 40 mg/kg) showed an increase of cell proliferation, however none of these differences were statistically significant (RVTL 20 mg/kg: 199 ± 6.6; RVTL 40 mg/kg: 220 ± 4.5; F_5,23_ = 6.12; *p* = 0.83, 0.086, respectively; Fig 1B and 1C) in comparison to control treated mice. However, the analysis of apoptotic cells revealed that RVTL (40 mg/kg) decreased the number of caspase 3 (active)-labeled cells (40%) in comparison to the control group (control: 187 ± 9.66 versus RVTL 40 mg/kg: 111 ± 4.41; F_5,23_ = 3.22; *p* = 0.027; Fig 1F and 1G). Thus, RVTL shows a pro-survival effect on the newborn cells in the dentate gyrus of female Balb/C mice at six months of age, which in general cannot be explained by an effect on precursor cell proliferation.

**Fig 2:**
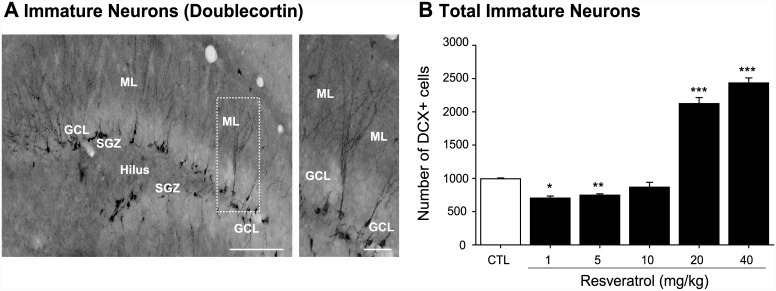
Resveratrol exhibits a dual effect on doublecortin-cells in female Balb/C mice at six months of age. (**A)** Representative micrograph of cells expressing doublecortin is shown. (**B)** Doublecortin (DCX)-labeled cells show significant decrease in mice treated with 1 and 5 mg/kg of RVTL (*p = 0.002, **p = 0.007, respectively). However, the highest doses of RVTL (20 and 40 mg/kg) significantly increase the absolute number of DCX-labeled cells (***p < 0.001). *n* = 4 mice per group. Data are expressed as the mean ± S.E.M. of the absolute number of cells. Results were analyzed with Student-Newman-Keuls after one-way ANOVA on ranks. Micrographs also show the granule cell layer (GCL), molecular layer (ML), subgranular zone (SGZ) and the hilus (H). Scale bar = 160 μm. Scale bar in amplification of panels A = 30 μm.

Additionally, we next evaluated the population of DCX-expressing cells, corresponding to the intermediate progenitor cells (type-2b and -3) and early post-mitotic stages of development (Fig 2A and 2B). Quantification at 14 days after treatment with RVTL revealed that the doses of 1 and 5 mg/kg significantly decreased (29% and 24%, respectively) the absolute number of DCX-cells in comparison to control mice (H = 25.10; control: 993 ± 12.1; RVTL 1 mg/kg: 704 ± 30.15; RVTL 5 mg/kg: 747 ± 20.40; q = 4.3, q = 5, respectively; *p* ≤ 0.001). However, the doses of 20 and 40 mg/kg of RVTL significantly increased (113% and 144%, respectively) the number of DCX-labeled cells (RVTL 20 mg/kg: 2122 ± 90.90; RVTL 40 mg/kg: 2433 ± 75.33; q = 4.58, q = 5.2, respectively; *p* ≤ 0.001) compared to the control mice. These results suggest that the lower doses of RVTL (1 and 5 mg/kg) affected DCX-cells, while higher doses of RVTL (20 and 40 mg/kg) favor the DCX-expressing intermediate population of cells.

### Resveratrol increases net hippocampal neurogenesis

**Fig 3:**
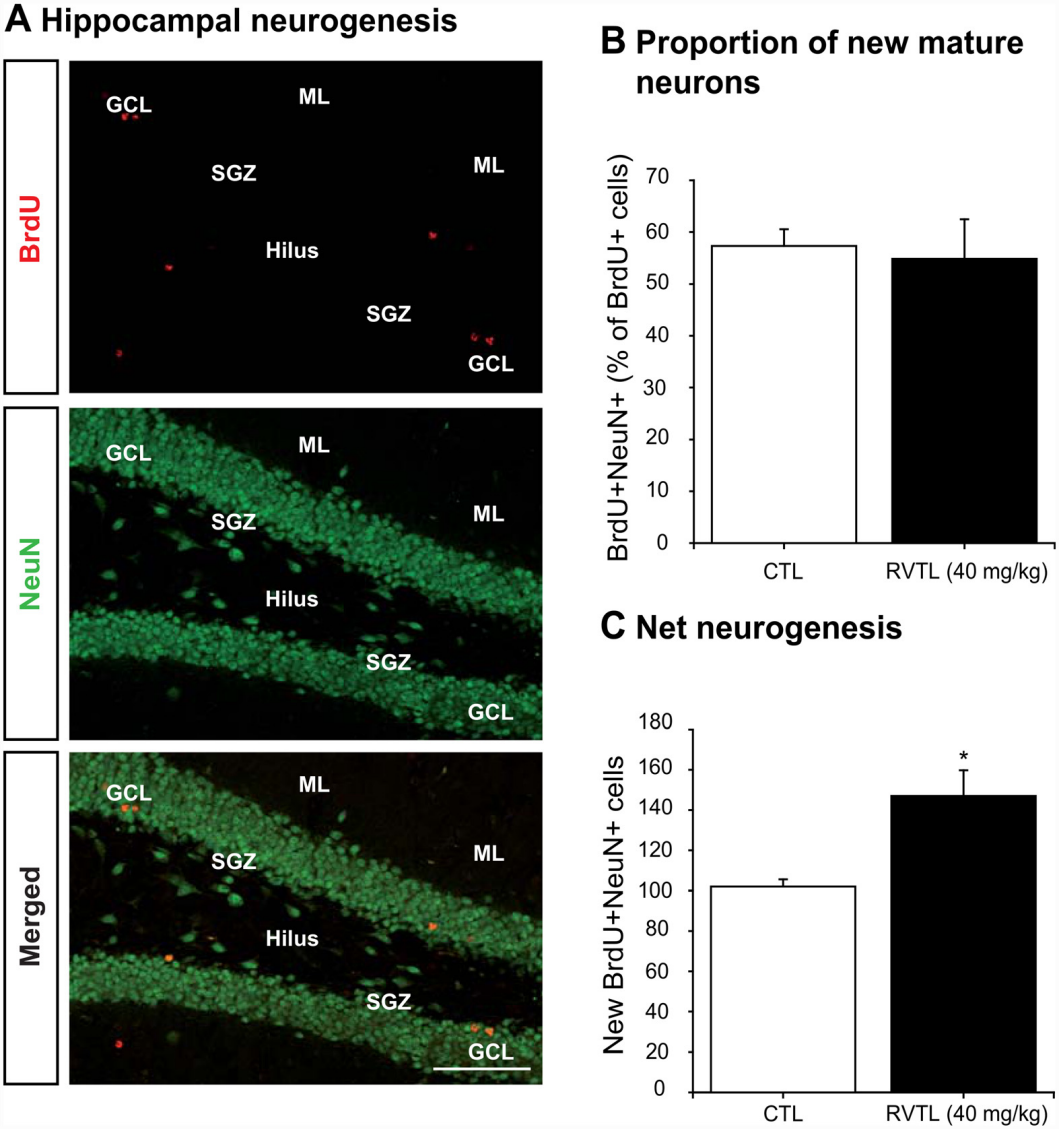
Resveratrol modifies net neurogenesis in female Balb/C mice at six months of age. (**A)** Proportion of mature new neurons was identified by BrdU- and NeuN-colabeling after 14 days of treatment. (**B)** Analysis shows that Resveratrol (RVTL; 40 mg/kg) did not modify the proportion of neuronal differentiation in female Balb/C mice at six months of age in comparison to the control (CTL) group. (**C)** Net neurogenesis was calculated as described in methods. Absolute numbers reflected that RVTL significantly increased net neurogenesis in female Balb/C mice at six months of age (*p = 0.015). *n* = 4 mice per group. Data are expressed as the mean ± S.E.M. of the absolute number of cells. Data were analyzed with the unpaired Student’s *t*-test. Micrographs also show the granule cell layer (GCL), molecular layer (ML), subgranular zone (SGZ) and the hilus (H). Scale bar = 180 μm.

Considering that the highest dose of RVTL showed consistent effects on cell survival, DCX-cells population and it decreased apoptosis, we further analyzed the phenotype of BrdU-labeled cells after treatment with RVTL (Fig 3). Phenotypic analysis revealed a similar proportion of newborn mature neurons in RVTL-treated (40 mg/kg) and in control mice (control: 57 ± 3.15 versus RVTL 40 mg/kg: 54 ± 7.58; t = 0.303; *p* = 0.77; Fig 3B). This implies that with increased survival the absolute number of new neurons significantly increased (44%) in the dentate gyrus of RVTL-treated mice (control: 102 ± 3.53 versus RVTL 40 mg/kg: 147 ± 12.77; t = -3.39; *p* = 0.015; Fig 3C).

### Resveratrol increases neuronal differentiation of adult hippocampal precursor cells without affecting cell proliferation in vitro with nanomolar concentrations

**Fig 4:**
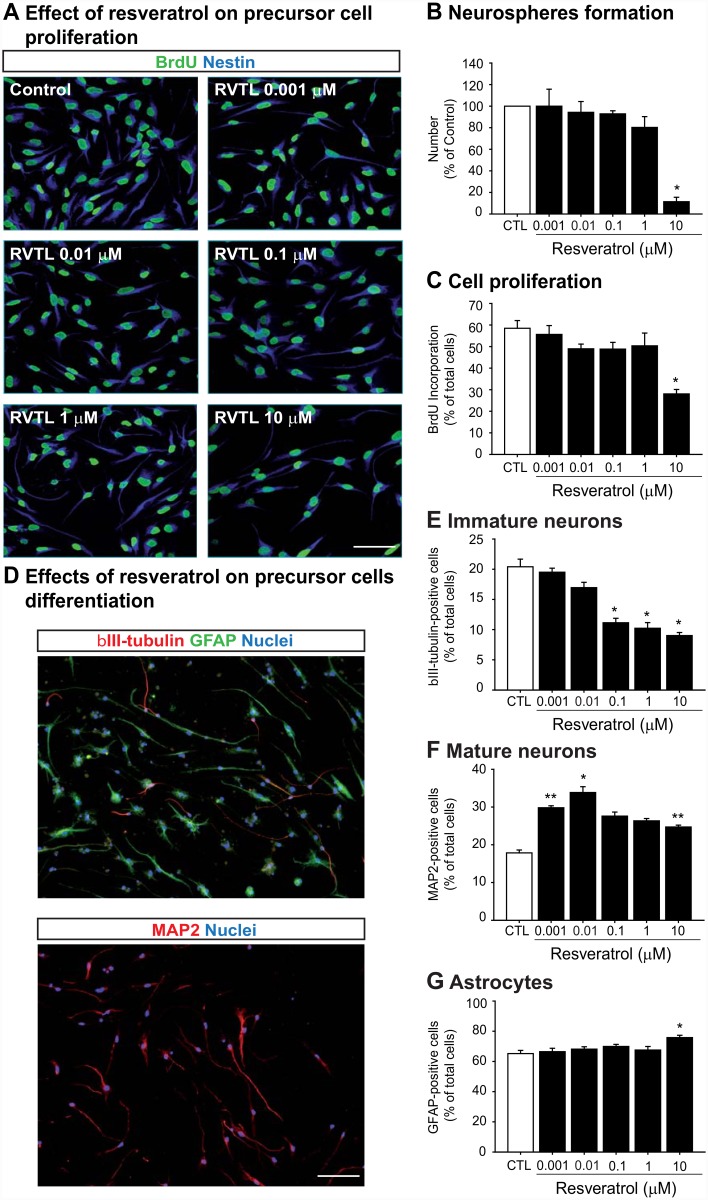
Resveratrol exhibits dual effects on adult hippocampal precursor cells *in vitro*. (**A-C)** Cell proliferation was determined as neurosphere formation (**B**) and in adherent hippocampal precursor cells by BrdU-incorporation (**A, C**). **(A)** Representative images of adherent hippocampal precursor cells expressing nestin that incorporated BrdU are shown. Scale bar = 40 μm. (**B)** Neurospheres quantification after 12 days of treatment with different concentrations of resveratrol (RVTL) showed a significant decrease with 10 μM (*p < 0.001). Data are expressed as the mean ± S.E.M. of percentage of control. Dunnet’s post hoc test was performed after one-way ANOVA. (**C)** Cell proliferation determined by BrdU incorporation showed a significant decrease with 10 μM RVTL (*p < 0.001). Data are expressed as the mean ± S.E.M. of percentage of total cells. Tukey’s post hoc test was performed after one-way ANOVA. (**D)** Representative images of βIII-tubulin-, GFAP- or MAP2-positive cells are shown. Scale bar = 100 μm. Cellular phenotypes were determined in adherent hippocampal precursor cells that underwent to differentiation with either the vehicle (CTL) or RVTL (0.001, 0.01, 0.1, 1 or 10 μM) for 5 days. (**E)** βIII-tubulin-positive cells were significantly decreased with 0.1, 1 and 10 μM RVTL (*p < 0.001), whereas (**F**) MAP2-positive cells were significantly increased with 0.001, 0.01 and 10 μM RVTL (**p = 0.02, *p = 0.047). (**G)** GFAP-positive cells derived from precursor cells showed that 10 μM RVTL significantly increased its proportion (*p = 0.014). Data are expressed as the mean ± S.E.M. of percentage of total Dapi cells. Tukey’s post hoc test was performed after one-way ANOVA. Experiments were done twice by quadruplicate.

In vitro precursor cells allow study of the direct effects of RVTL in a controlled environment (Fig 4). First, we analyzed whether RVTL might induce the formation of neurospheres as an index of precursor cell activity and proliferative expansion (Fig 4B). The number of neurospheres generated from cells cultured with nanomolar (0.001–0.1 μM) or 1 μM concentrations of RVTL did not show significant changes, whereas the highest concentration of RVTL (10 μM) significantly decreased (∼88%) the number of neurospheres formed in comparison to the control treated cells (*p* < 0.001). Similarly to neurospheres, in adherent neural hippocampal precursor cells 10 μM of RVTL also decreased proliferation by ∼30% as assessed by the incorporation of BrdU (F_5,37_ = 10.30; *p* < 0.01; Fig 4A and 4C).

Next, we analyzed whether RVTL might induce differentiation from precursor cells in vitro (Fig 4D–4G). Phenotypic analysis of cultures differentiated in the presence of different concentrations of RVTL (0.001–10 μM) showed that the proportion of βIII-tubulin-positive immature neurons was decreased in cells cultured with concentrations ranging from 0.1 to 10 μM (F_5,47_ = 33.41; *p* < 0.001; Fig 4E). Moreover, the proportion of mature neurons generated from precursor cells showed that nano- and micro-molar concentrations of RVTL (0.001; 0.01 or 10 μM; F_5,53_ = 11.84; *p* = 0.02, 0.047; 0.02) significantly increased the proportion of MAP2ab-positive neurons (Fig 4F). The proportion of GFAP-positive astrocytes was also significantly increased in cells cultured with 10 μM of RVTL compared to un-treated controls (F_5,47_ = 3.92; *p* = 0.014; Fig 4G). These results suggest that while nanomolar concentrations of RVTL did not affect cell proliferation they increased the number of neurons generated from the precursor cells in vitro.

### Resveratrol increases the number of dendritic spines

**Fig 5:**
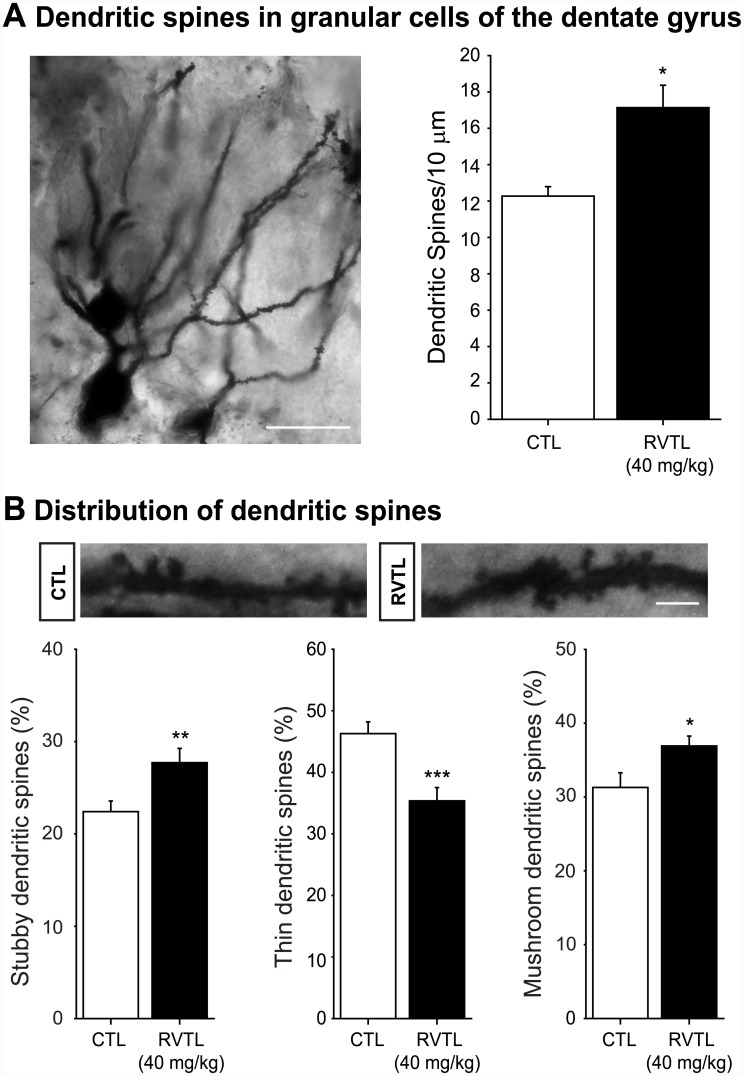
Resveratrol increases dendritic spines in granular cells of the dentate gyrus in female Balb/C mice at six months of age. (**A)** Micrograph depicts granular cells with primary and secondary dendrites in the dentate gyrus (DG). Dendrite projections reach the molecular layer and show some protuberances corresponding to dendritic spines. Scale bar = 30 μm. Number of dendritic spines along 10 μm in secondary dendrites was higher in mice treated with resveratrol (RVTL; 40 mg/kg) than control treated mice (*p = 0.001). Data are expressed as the mean ± S.E.M. and were analyzed applying an un-paired Student’s *t*-test. (**B)** Representative micrographs of dendritic spines of granular cells derived from control (CTL) or RVTL-treated mice are shown. Scale bar = 5 μm. Proportion of the different types of dendritic spines showed significant increases in stubby- and mushroom-shape dendritic spines in RVTL-treated mice (**p = 0.011, *p = 0.026, respectively), whereas the thin-shape dendritic spines were higher in CTL treated mice (***p < 0.001). Data are expressed as the mean ± S.E.M. of the proportion for each dendritic spine-shape. Data were analyzed with the unpaired Student’s *t*-test. *n* = 4–5 mice per group.

In order to further extend our study to another aspect of plasticity taking place in the dentate gyrus, we analyzed the number of dendritic spines on the secondary dendrites of granule cells (Fig 5A). Mice treated with RVTL (40 mg/kg) showed a greater number (plus ∼35%) of dendritic spines than the control group (control: 12.26 ± 0.52 versus RVTL 40 mg/kg: 17.13 ± 1.23; t = -4.86; *p* = 0.001). The comparison of treatment within every category of dendritic spines revealed that RVTL (40 mg/kg) significantly increased the proportion of mushroom-shape spines (control: 31 ± 1.99% versus RVTL 40 mg/kg: 36 ± 1.33%; t = -2.34; *p* = 0.026; Fig 5B) and stubby-shape spines (control: 22 ± 1.15% versus RVTL 40 mg/kg: 27 ± 1.55%; t = -2.74; *p* = 0.011; Fig 5B). The proportion of thin spines in contrast was decreased in mice treated with RVTL (control: 46 ± 1.90% versus RVTL 40 mg/kg: 35 ± 2.15%; t = 3.79; *p* < 0.001; Fig 5B). The increased number of dendritic spines and greater proportion of mushroom-shape spines indicates that RVTL promotes spine formation and maturation in granule cells of the dentate gyrus.

### Resveratrol improves behavioral performance

**Fig 6:**
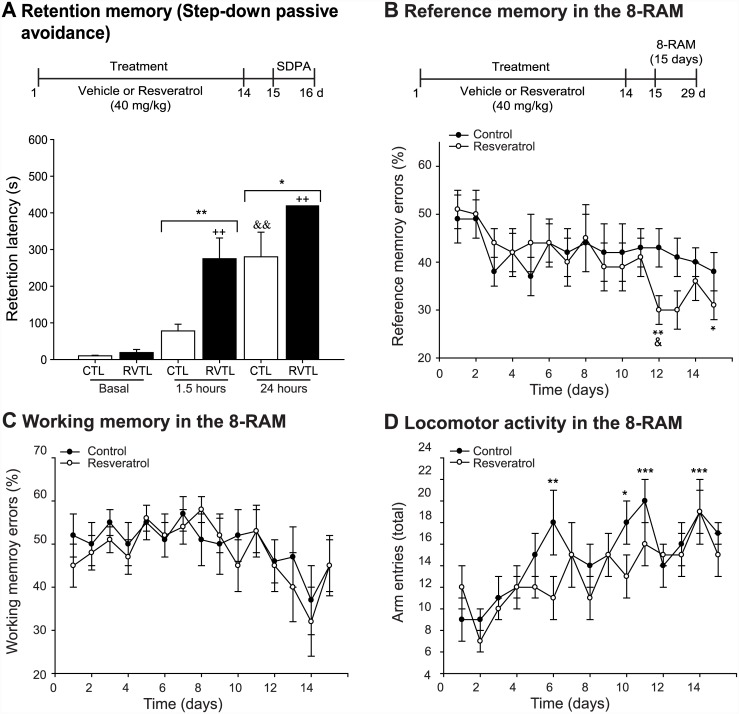
Resveratrol improves performance of female Balb/C mice at six months of age in the SDPA. (**A**) Different cohorts of mice were used to test their improve performance in the step down passive avoidance (SDPA). One day after the end of the treatment the SDPA was performed and tested 1.5 (day 15) and 24 hours (day 16) after training. Control (CTL) or resveratrol (RVTL; 40 mg/kg) treated mice were tested in the step-down passive avoidance test. Resveratrol treated mice showed significant increase in the retention latency to move into the dark side of the chamber than the CTL group at 1.5 (**p < 0.001) and 24 hours (*p = 0.011) after basal test. *n* = 8 per group Along the exposure sessions mice treated with RVTL (40 mg/kg) showed an increase in the retention latency in comparison to the basal test (^++^ *p* < 0.001). However, control mice just showed significant increase latency at 24 hours in comparison to the basal test (^&&^ *p* < 0.001). Data are expressed as the mean ± S.E.M. of the retention latency parameter. Tukey’s post hoc test was performed after repeated measures two-way ANOVA. (**B**) Eight-radial arm maze (8-RAM) was performed after 14 days of treatment with RVTL (40 mg/kg) and the training was followed for 15 days (day 15–29). Also mice treated with RVTL (40 mg/kg) showed a decrease percentage of reference memory errors along the training in the eight-radial arm maze (8-RAM). Significant differences were observed at the day 12 (**p = 0.005) and 15 (*p = 0.015) in comparison to the first session (day 1); whereas control treated mice did not show significant differences (*p* = 1). In addition, differences between RVTL and the respective session of CTL group were analyzed applying unpaired Student’s *t*-test reflecting that at day 12 exist a significant difference (^**&**^ *p* = 0.041) between groups. (**E**) Percentage of working memory errors in both groups was not changed (*p* = 1). (**F**) The total number of entries to the eight arms of the maze was not different along the sessions within RVTL (*p* ≤ 1), however the control group showed differences respective to the first session (day_6_ **p = 0.008; day_10_ *p = 0.015, days_11,14_ ***p ≤ 0.001), but not between treatment per sessions (*p* = 0.216). Data are expressed as the mean ± S.E.M. *n* = 12 mice per group.

We next asked whether the positive effects of RVTL on adult hippocampal neurogenesis and spine formation occurred in parallel with effects on associative memory, here as reflected by the retention latency in the step-down passive avoidance task (SDPA; Fig 6A). The assessment of retention latency 1.5 hours after basal test revealed a statistically significant difference in RVTL-treated mice compared to controls (control: 78 ± 18 seconds versus RVTL 40 mg/kg: 275 ± 67.3 seconds; F_1,44_ = 15.72; *p* < 0.001). A similar effect was also seen at 24 hours after the basal test (control: 280 ± 67.3 seconds versus RVTL 40 mg/kg: 418 ± 0.5 seconds; F_1,44_ = 15.72; *p* = 0.011). Moreover, along the exposure sessions within RVTL, mice showed a statistically significant difference in the retention latency at 1.5 and 24 hours in comparison to the basal test (F_2,44_ = 40.90; *p < 0*.*001*); whereas the comparison of exposure sessions within control-treated mice showed a significant difference at 24 hours (F_2,44_ = 40.90; *p < 0*.*001*) in comparison to the basal test.

Additionally, we analyzed the performance of RVTL-treated mice in the eight-radial arm maze (8-RAM) during the next 15 days after the end of the treatment (Fig 6B–6D). In this test, both, control and RVTL-treated mice showed a progressive decrease in reference memory errors during training (Fig 6B). Analysis of the effect of treatment per session reflected a significant decrease in percentage of reference memory errors in RVTL-mice on day 12 in comparison to their respective control (t = 2.109; *p* = 0.041; Fig 6B). Moreover, along the consecutive daily sessions within RVTL, mice showed a decrease percentage of reference memory errors and statistically significant differences were observed on days 12 and 15 (F_14,289_ = 2.59; *p* = 0.005, 0.015, respectively) in comparison to their first session (day 1); whereas the comparison of daily sessions within control-treated mice did not show significant differences (F_14,289_ = 2.59; *p* = 1). In contrast, working memory errors (F_14,289_ = 3.66; *p* = 1; Fig 6C) were not significantly different. In addition, the total arms entries of the maze, an index of locomotor activity, did not show differences along the consecutive daily sessions within RVTL group (F_14,289_ = 6.39; *p* ≤ 1;). However, the comparison of daily sessions within the control group showed differences related to their respective first session (D_6_ = 0.008; D_10_ = 0.015; D_11_ < 0.001; or D_14_ = 0.001), but not between treatment per session (F_14,289_ = 1.30; *p* = 0.216; Fig 6D), Together, these results suggest that RVTL strongly increased performance in the SDPA and had little effect in the 8-RAM test, suggesting a beneficial effect of RVTL on a specific type of learning after 14 days of treatment with RVTL.

### Resveratrol increases the Akt and PKC phosphorylation

**Fig 7:**
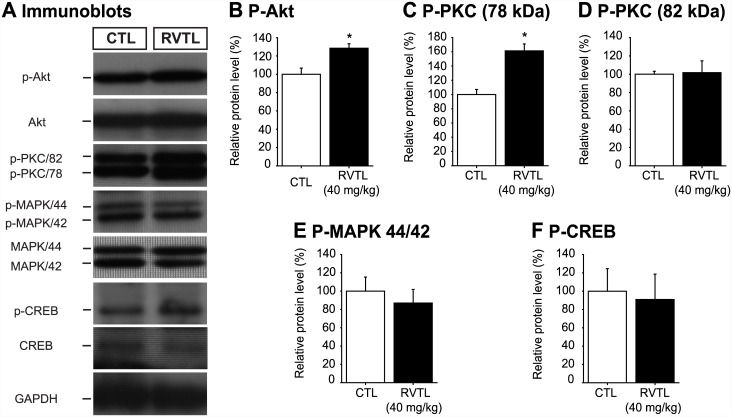
Resveratrol increases the levels of phosphorylated- Akt and—PKC proteins in the hippocampus of female Balb/C mice at six months of age. (**A)** Representative immunoblots of the phosphoproteins analyzed in the hippocampal protein extracts of mice treated with resveratrol (RVTL, 40 mg/Kg) are shown. (**A-C)** Resveratrol treated mice showed significant increase in the levels of phospho-Akt (*p = 0.017) and phospho-PKC (78 kDa) (*p = 0.002), whereas the levels of phospho-PKC (82 kDa, *p* = 0.91; **D**), phospho-MAPK 44/42 (*p* = 0.56; **E**) and phospho-CREB (*p* = 0.82; **F**) did not show significant changes. Data were analyzed with the unpaired Student’s *t*-test. *n* = 4–5 mice per group.

In order to get first insight about a possible signaling mechanism involved in the effects of RVTL on neuroplasticity, we looked at the relative levels of key signaling proteins known to be involved in regulating cell proliferation, survival and dendritic spine formation in the total protein extracts of hippocampus (Fig 7A). Mice treated with RVTL (40 mg/kg) showed a significant increase in the phosphorylated form of protein kinase B (also known Akt; ~28%) in comparison to control treated mice (control: 100 ± 7.9% versus RVTL 40 mg/kg: 128 ± 4.85%; t = -3.96; *p* = 0.017; Fig 7B). A similar effect was seen in the levels of one of the two identified phosphorylated forms of protein kinase C (PKC 78 kDa; control: 100 ± 7.13% versus RVTL 40 mg/kg: 161 ± 9.62%; t = -4.87; *p* = 0.002; Fig 7C). However, we did not find changes in the levels of the active isoform of PKC at 82 kDa; (t = -0.11, *p* = 0.91; Fig 7D), in the levels of the phosphorylated forms of the mitogen activated protein kinases 44/42 (MAPK 44/42; t = 0.61, *p* = 0.56; Fig 7E) and of cAMP response element-binding protein (CREB; t = -0.24, *p* = 0.81; Fig 7F). These results might suggest the involvement of Akt and PKC (78 kDa) in the mechanism by which RVTL modulates brain structural changes such as hippocampal neurogenesis and dendritic spines.

## Discussion

In the present study, we have explored the complex effects of RVTL on neuroplasticity including in it the generation of new neurons and the density and morphology of dendritic spines on hippocampal granule cells in female Balb/C mice at six months of age. In addition, we found that RVTL-treated mice showed improvement performance in behavioral tests related to memory formation.

Basic and preclinical studies have already provided information concerning the broad effects of RVTL on the organisms related to metabolism and obesity, cardiovascular function, cancer and, most notably, life-span [39–41].

Studies in animal models of Alzheimer’s disease showed that RVTL prevented cognitive-learning impairment [42] and hippocampal neurodegeneration [43]. Common among these conditions is that aging is a key risk factor for the development of neurodegeneration and that RVTL may act as an anti-aging molecule to counteract age-related risks (see, for example, discussion in [44]).

In this context, it is somewhat surprising that in this context adult neurogenesis has not attracted more attention. In a murine model of chronic fatigue, the oral administration of RVTL (40 mg/kg) rescued the population of proliferative cells [23], whereas in prenatally stressed rats the administration of RVTL (10 mg/kg) favored the recovery of the DCX-population [22].

Treating calcitonin gene related peptide (CGRP) knockout mice with 20 mg/kg RVTL reportedly caused an increase in cell proliferation; however, this result may also reflect the short-term survival effects after 5 days of cumulative BrdU injections. Moreover, RVTL increases BrdU/Calbindin colabeled cells in CGRP knockout mice [24]. By and large these results agree with our findings here, even though our data concerning the effects of RVTL on proliferation and, at lower doses, on DCX-cells do not allow for a definitive conclusion. Nevertheless, in conjunction with the DCX and caspase 3 (active) findings, our observations also support the view that RVTL acts on precursor cell stages in the course of adult neurogenesis to affect multiple aspects of development, including the promotion of cell survival.

However, there are contradictory findings in the literature as well. In one study, mice that were treated with RVTL by intraperitoneal administration (1 or 10 mg/kg) showed a decrease in cell proliferation, survival, and the numbers of DCX-positive cells and new neurons. In the same study, but at the precursor cell level, micromolar concentrations of RVTL (20 to 50 μΜ) decreased proliferation of embryonic cerebral cortical and C17.2 precursor cells [20]. Moreover, hippocampus-derived precursor cells that were cultured with different concentrations of RVTL (2 to 4 μΜ) showed a decrease in the number of βIII-tubulin neurons that were derived from differentiated adult neurospheres [21]. Similarly, our study revealed that the in vitro addition of 10 μΜ of RVTL affected cell proliferation.

These discrepancies regarding the effects of RVTL on adult hippocampal neurogenesis may be due to several factors, such as the strain of mice, the duration of treatment, the timing and mode of labeling of proliferative cells and the age of mice at the time of analysis. Thus, depending upon the dose, the route of administration and the concentration of RVTL that reaches the brain, it is possible to observe a positive regulation of the neurogenic process. Here, the survival of newborn cells in female Balb/C mice at six months of age was of central importance, particularly because our general hypothesis is that adult hippocampal neurogenesis provides a “neurogenic reserve” that allows for the maintenance of cognitive flexibility in older age. We showed that the oral administration of RVTL (40 mg/kg) decreased apoptosis and increased cell survival, the population of DCX-cells and, consequently, increased hippocampal neurogenesis in female Balb/C mice at six month of age without affecting cell proliferation after 14 days of treatment. Interestingly cellular proliferation was not affected at the precursor level in adherent cells or in neurospheres that were derived from the dentate gyrus that were cultured with nanomolar concentrations of RVTL. Unfortunately, we did not analyze the presence of resveratrol in the lysates of dissected hippocampus, however the pharmacokinetic studies have reported a very low quantity of RVTL in the brains of rodents after 14 days of oral administration [16]. Thus, it is possible that nanomolar concentrations of RVTL favor hippocampal neurogenesis in female Balb/C mice at six months of age, whereas micromolar concentrations impair the neurogenic process in the hippocampus.

In addition to the effects of RVTL on hippocampal neurogenesis, this compound increased the number of dendritic spines and the proportion of more mature dendritic spines (mushroom shape) in granular cells of the dentate gyrus, which may suggest its beneficial effect on the learning and memory processes [45]. Our study also showed that the treatment with RVTL improved performance of female Balb/C mice at six months of age in tests that were related to learning and memory such as the SDPA and the 8-RAM. Interestingly, RVTL-treated mice showed better performance in the SDPA than in the 8-RAM. The differences in these results could be related to the type of learning and memory assessed with these tests because the SDPA and the 8-RAM recruit different behavioral, neural, and cognitive demands (i.e., [46]). Thus, in accordance with our results RVTL improved fear- and spatial- memories, the former more significantly than the latter. Thus, RVTL produced stronger effects in acute responses (SDPA) than in long-lasting acquisition procedures (8-RAM). Nevertheless, our data did not discard any effect on learning- and memory-related paradigms that could be produced by longer treatment with RVTL. Nevertheless, our data demonstrated that concomitantly with cell survival and hippocampal neurogenesis, RVTL modulates neuroplasticity at the age of 6 months, a time when physiological levels of adult neurogenesis have declined to very low baseline levels (i.e., [27, 28, 30]).

One main focus of research has been to observe the effects of RVTL on the so-called longevity gene Sirt1, which may imply the promotion of epigenetic effects by RVTL [47]. Numerous other direct or indirect mechanisms have been proposed at several levels, which may ultimately yield an epigenetic cause. Initially, RVTL was promoted as an antioxidant; however despite growing insight into its molecular links, such as to the sirtuin genes, its exact mechanisms of action remain elusive. In a recent review, Kulkarni and Cantó concluded that *“The intrinsic anti-oxidant capacity of the resveratrol molecule and its ability to trigger the activation/repression of a wide range of membrane receptors*, *kinases and other enzymes have turned the quest for a molecular mechanism of action into an epic task*.*”*[48].

Accordingly, the mechanisms by which RVTL may promote neuroplasticity are unknown; however, recent studies have proposed that AMP-activated protein kinase and Sirt-1 [17, 18] have beneficial participation on RVTL in neurodegenerative diseases models [16]. Additionally, it is known that the neuroprotective effects of RVTL are partially mediated by the activation of the γ isoform of protein kinase C, which exhibits a molecular weight of approximately 78 kDa [49]. Here, we found an increased level of phospho-PKC (78 kDa) in the hippocampal lysates of mice that were treated with RVTL (40 mg/kg). Members of the protein kinase C family have been involved in several biological events, such as cell proliferation and survival, as well as in the reorganization of the cytoskeleton [50–53]. Moreover, we found an increase in the active form of the Akt protein that is involved in both the dendrite maturation and the promotion of cell survival [54]. Our study suggests that RVTL may involve phospho-PKC and phospho-Akt increases to induce neuroplastic changes that consequently improve performance in tests that are related to the learning and memory processes in female Balb/C mice of six months of age. Nevertheless, our results cannot discard the influence of RVTL on the epigenetic modifications, which may be relevant for the positive effects that were observed on neuroplasticity and that could be modulated through Sirt-1, as was reported in other models (for review see [9]). The possible mechanism by which RVTL modulates neuroplasticity deserves further investigation.

The findings of our preclinical study provide additional evidence to support the hypothesis that RVTL exerts beneficial effects on the brain; however, it is important to study the mechanism that is involved in the modulation of neuroplasticity to better understand the value of the natural compounds found in the diet, such as RVTL, that could help to delay the presence of neuroplastic and cognitive alterations during normal aging. In this regard, a recent clinical study reported that RVTL improved memory performance and hippocampal functional connectivity in healthy older adults (i.e., [10]), however, RVTL was supplemented in combination with another flavonoid, quercetin, which also is known to influence neurogenesis. For this reason, these results are difficult to use in the interpretation of the effect of RVTL alone on neuroplasticity and memory.

Due to the complex nature of the effects of RVTL, it is necessary to perform controlled clinical studies to decipher the effect of this flavonoid on neuroplasticity [55]. Thus, our present data support the idea that RVTL is a compound that has interesting implications for strategies that seek to improve brain plasticity in the context of aging or brain disease. Nevertheless, our data indicate that the role of RVTL in the context of adult neurogenesis and other types of neuroplasticity within an improvement in learning and memory is worth pursuing.
